# Multimodal Management of Isolated Pulmonary Metastasis From Choroidal Melanoma: A Case Report

**DOI:** 10.7759/cureus.116349

**Published:** 2026-09-16

**Authors:** Francisca Abreu, Rita Amorim e Costa, Tiago Figueiredo, Paula Ferreira, Sara Coelho

**Affiliations:** 1 Department of Oncology, Unidade Local de Saúde do Alto Ave, Guimarães, PRT; 2 Department of Oncology, Instituto Português de Oncologia do Porto Francisco Gentil, Porto, PRT; 3 Department of Radiotherapy, Instituto Português de Oncologia do Porto Francisco Gentil, Porto, PRT

**Keywords:** choroidal melanoma, extrahepatic metastases, immune-checkpoint inhibitor (ici), nf1 mutation, stereotactic ablative body radiotherapy

## Abstract

Choroidal melanoma (CM) is the most common type of uveal melanoma (UM), which represents the most common primary intraocular malignancy in adults, and it is characterized by a high risk of distant metastasis despite adequate local treatment. While the liver is the predominant site of relapse, isolated pulmonary involvement can occur, which represents an uncommon clinical phenotype. We report the case of a 47-year-old woman with late isolated lung metastasis from previously treated CM, diagnosed eight years after primary treatment with episcleral brachytherapy. Comprehensive genomic profiling revealed a high tumor mutational burden (TMB) of 16.5 mutations/Mb and a somatic nonsense neurofibromin 1 (NF1) variant (p.R2637*), in the absence of B-Raf proto-oncogene, serine/threonine kinase (BRAF) mutation or neuroblastoma RAS viral oncogene homolog (NRAS) mutation, findings atypical for UM. The patient was human leukocyte antigen A02:01 (HLA-A02:01) negative, precluding tebentafusp therapy. She received dual immune checkpoint blockade (nivolumab 1 mg/kg plus ipilimumab 3 mg/kg), experiencing manageable cutaneous and endocrine toxicities. However, she subsequently experienced disease progression. Subsequent management included stereotactic body radiotherapy (SBRT) (45 Gy in five fractions) and cytotoxic chemotherapy with carboplatin area under the curve 5 (AUC 5) and paclitaxel (175 mg/m^2^), with dose reduction due to toxicity. At 18-month follow-up, the patient remains in surveillance with stable disease.

This case underscores the utility of expanded molecular profiling in metastatic choroidal melanoma (mCM) to identify predictive biomarkers and emerging therapeutic targets in a disease with limited standard-of-care options.

## Introduction

Choroidal melanoma (CM) is a distinct biological entity from cutaneous melanoma, carrying a high risk of distant relapse despite adequate local treatment, such as enucleation or plaque brachytherapy [1-3]. Approximately 50% of patients eventually develop metastatic disease, which exhibits a marked tropism for the liver (90-93%) [2-3]. Isolated or lung-predominant metastases occur in only 10% of cases and represent an uncommon clinical phenotype associated with significantly longer survival (median 17 months) compared to hepatic-dominant disease (median 7-11 months) [4].

Standard systemic therapies, particularly immune checkpoint inhibitors (ICIs), have demonstrated modest efficacy in metastatic choroidal melanoma (mCM) compared to cutaneous melanoma [3]. Dual ICI therapy with nivolumab and ipilimumab typically yields overall response rates (ORR) of 10 to 18% and median progression-free survival of about 5 months [5-7]. Furthermore, combined immune checkpoint blockade carries a substantial risk of immune-related adverse events (irAEs) with grade ≥3 according to the Common Terminology Criteria for Adverse Events version 5.0 (CTCAE v5.0), occurring in approximately 40-60% of cases in recent real-world and trial cohorts [5-8]. However, advances in next-generation sequencing (NGS) have enabled the identification of rare molecular subgroups, including tumors harboring neurofibromin 1 (NF1) mutations or high tumor mutational burden (TMB), which may have potential therapeutic implications [7]. High TMB, in particular, has been associated with increased tumor immunogenicity and may predict benefit from ICIs in selected tumor types.

In this study, we present a case of isolated pulmonary metastasis from CM with an atypical molecular profile (a high TMB and NF1 mutation), treated with dual checkpoint blockade, complicated by cutaneous and endocrine irAEs, and followed by local ablative therapy and chemotherapy.

## Case presentation

Initial presentation and diagnosis (2016)

A 47-year-old woman with a history of chronic gastritis (*Helicobacter pylori *positive) and degenerative osteoarticular disease presented to the emergency department in 2016 with a spontaneous right vitreous hemorrhage, with no history of trauma or other apparent precipitating cause. The chronological sequence of the patient's clinical course, molecular characterization, treatments, and outcomes is summarized in Figure 1. She had no family history of melanoma or other malignancies.

**Figure 1:**
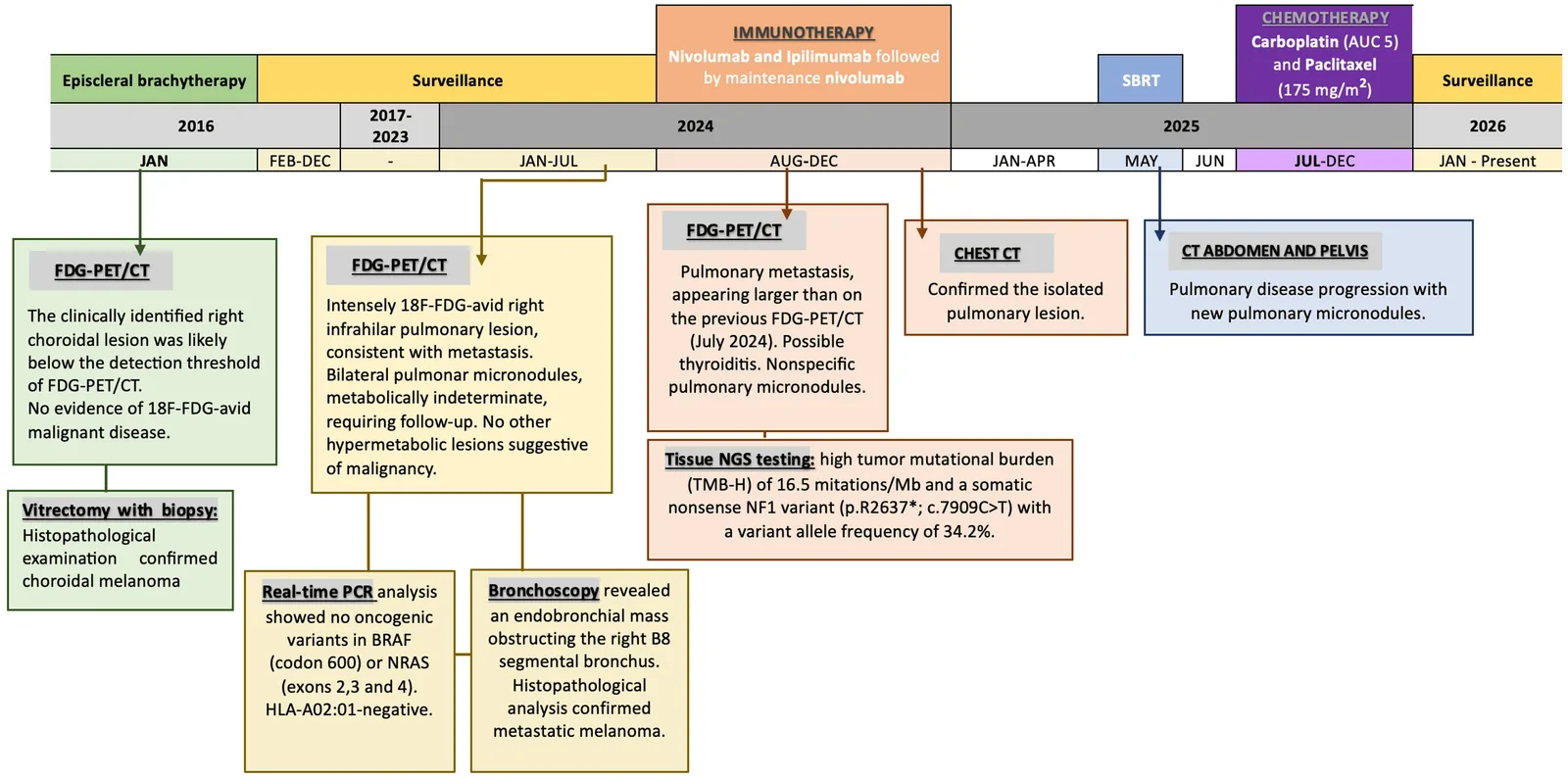
Timeline of the patient's clinical course Timeline summarizing the patient's clinical course, including initial diagnosis, molecular characterization, systemic treatments, stereotactic body radiotherapy, and disease evolution.

Ophthalmologic examination revealed an inferior, dark subretinal lesion associated with hemorrhage and multiple retinal breaks. Staging with fluorodeoxyglucose positron emission tomography/computed tomography (FDG-PET/CT) showed no evidence of distant metastatic disease (Figure 2). The patient subsequently underwent vitrectomy with biopsy of the lesion, and histopathological examination established the diagnosis of CM. The patient underwent successful primary treatment with episcleral brachytherapy with iodine-125 and remained under regular oncological surveillance for eight years without evidence of recurrence.

**Figure 2:**
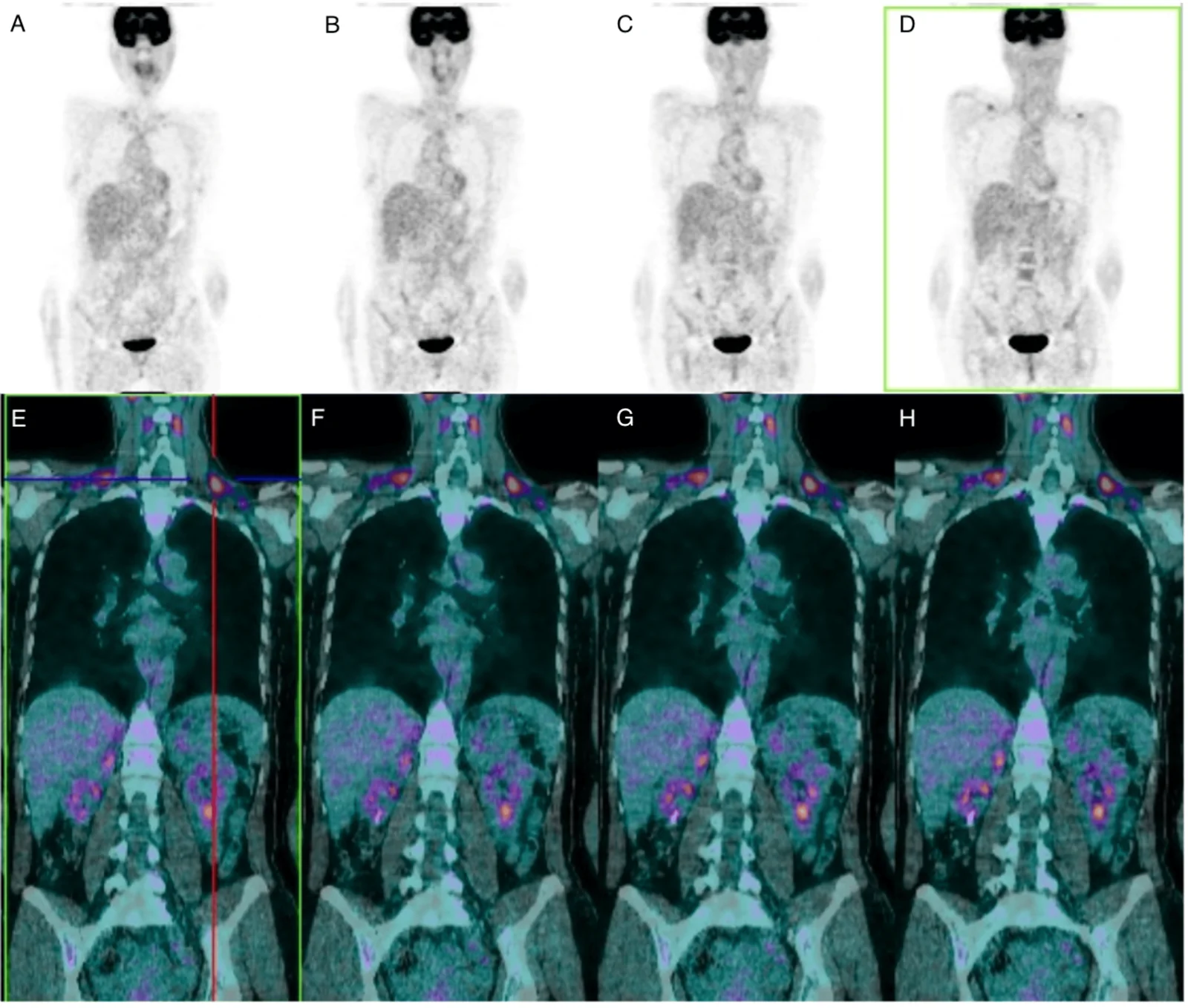
Baseline FDG-PET/CT at initial diagnosis Baseline FDG-PET/CT performed at initial diagnosis, showing no evidence of distant metastatic disease. (A-D) FDG-PET images and (E-H) fused FDG-PET/CT images. The clinically identified right choroidal lesion was likely below the detection threshold of FDG-PET/CT. Therefore, no arrow is shown. FDG-PET/CT: fluorodeoxyglucose positron emission tomography/computed tomography

Detection of metastatic disease (2024)

In 2024, following a respiratory infection, a chest radiograph revealed a suspicious pulmonary opacity. Subsequent FDG PET/CT demonstrated a 30 × 30 mm hypermetabolic solid lesion in the right peribronchial region, below the bifurcation of the bronchus intermedius, consistent with malignancy, along with multiple bilateral pulmonary micronodules (Figure 3). Bronchoscopy with biopsy was performed and revealed an endobronchial mass obstructing the right B8 segmental bronchus. Histopathological analysis confirmed metastatic melanoma with endobronchial involvement.

**Figure 3:**
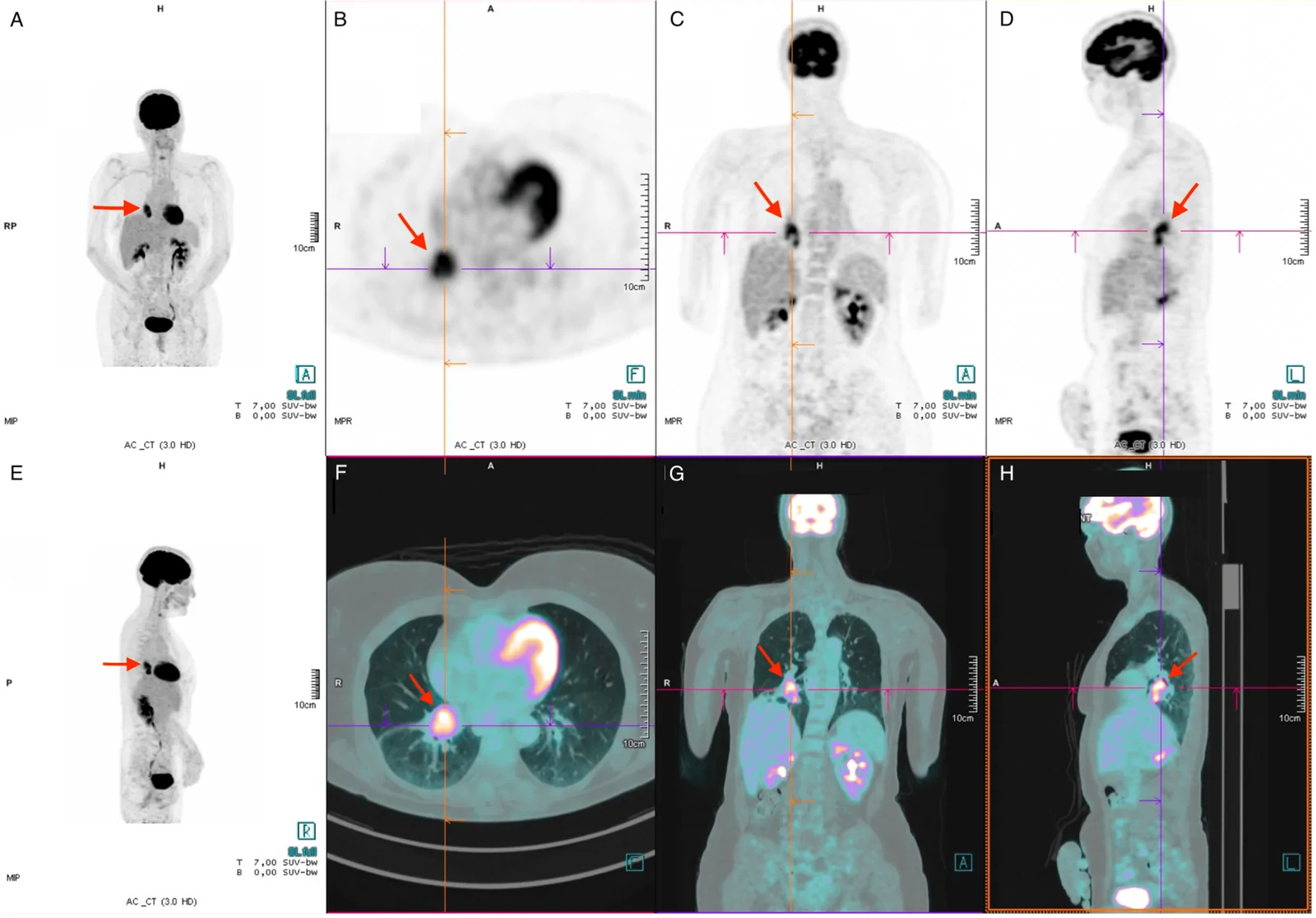
FDG-PET/CT showing pulmonary metastatic disease FDG-PET/CT demonstrating a hypermetabolic solid lesion in the right peribronchial region, below the bifurcation of the bronchus intermedius, consistent with metastatic disease. (A, E) FDG-PET maximum-intensity projection images. (B-D) FDG-PET images in axial, coronal, and sagittal views, respectively. (F-H) Fused FDG-PET/CT images in axial, coronal, and sagittal views, respectively. The arrows indicate the detected lesion described in the text. FDG-PET/CT: fluorodeoxyglucose positron emission tomography/computed tomography

Clinically, following resolution of a respiratory tract infection, the patient remained asymptomatic and maintained an excellent functional status, with an Eastern Cooperative Oncology Group performance status (ECOG PS) of 0. The remaining staging workup, including brain magnetic resonance imaging (MRI) with contrast and abdominal MRI with gadolinium, revealed no evidence of additional secondary lesions, specifically hepatic or cerebral metastases. Baseline laboratory values showed results within normal limits.

Molecular characterization

To guide systemic therapeutic decision-making, comprehensive molecular and genetic characterization was performed. Initial real-time polymerase chain reaction (PCR) analysis showed no oncogenic variants in B-Raf proto-oncogene, serine/threonine kinase (BRAF) (codon 600) or neuroblastoma RAS viral oncogene homolog (NRAS) (exons 2, 3, and 4). Furthermore, the patient was identified as human leukocyte antigen A02:01 (HLA-A02:01) negative, precluding eligibility for tebentafusp. Subsequent comprehensive genomic profiling was performed using the 523-gene TruSight Oncology 500 (TSO500) targeted hybrid-capture next-generation sequencing assay (Illumina Inc., San Diego, CA, USA). Sequencing was performed on an Illumina NextSeq platform, with secondary analysis using the TSO500 v2.2 Local App and a customized analysis pipeline within the PierianDx Clinical Genomics Workspace. Laboratory reported variants only in regions with a minimum sequencing coverage >100×. The analysis revealed a high TMB of 16.5 mutations/Mb and a somatic NF1 nonsense variant (NM_001042492.2:c.7909C>T; p.Arg2637Ter [R2637*]) with a variant allele frequency of 34.2%. Microsatellite status was stable. No G protein subunit alpha Q (GNAQ) or G protein subunit alpha 11 (GNA11) mutations were identified.

Treatment course and outcomes

In August 2024, the patient initiated dual immune checkpoint blockade with nivolumab (1 mg/kg) and ipilimumab (3 mg/kg) every three weeks for four cycles, followed by maintenance nivolumab (480 mg every four weeks). Shortly after the first cycle, she developed grade 2 cutaneous toxicity, characterized by maculopapular rash and pruritus, alongside grade 1 immune-mediated thyroiditis, associated with fatigue, both according to CTCAE v5.0. Endocrinologic evaluation confirmed immune-mediated thyroiditis, and levothyroxine 50 µg daily was initiated. These irAEs were managed with supportive medical therapy, allowing the treatment to continue without interruption.

After completing four cycles of induction therapy followed by six cycles of maintenance nivolumab, a FDG-PET/CT scan in November 2024 demonstrated metabolic progression of the pulmonary lesion (Figure 4). A subsequent chest CT confirmed progression of the pulmonary lesion (Figure 5). Given the oligoprogressive nature of the isolated lung lesion, the patient underwent stereotactic body radiotherapy (SBRT) between March 3 and March 12, 2025, receiving a total dose of 45 Gy in five fractions via volumetric modulated arc therapy.

**Figure 4:**
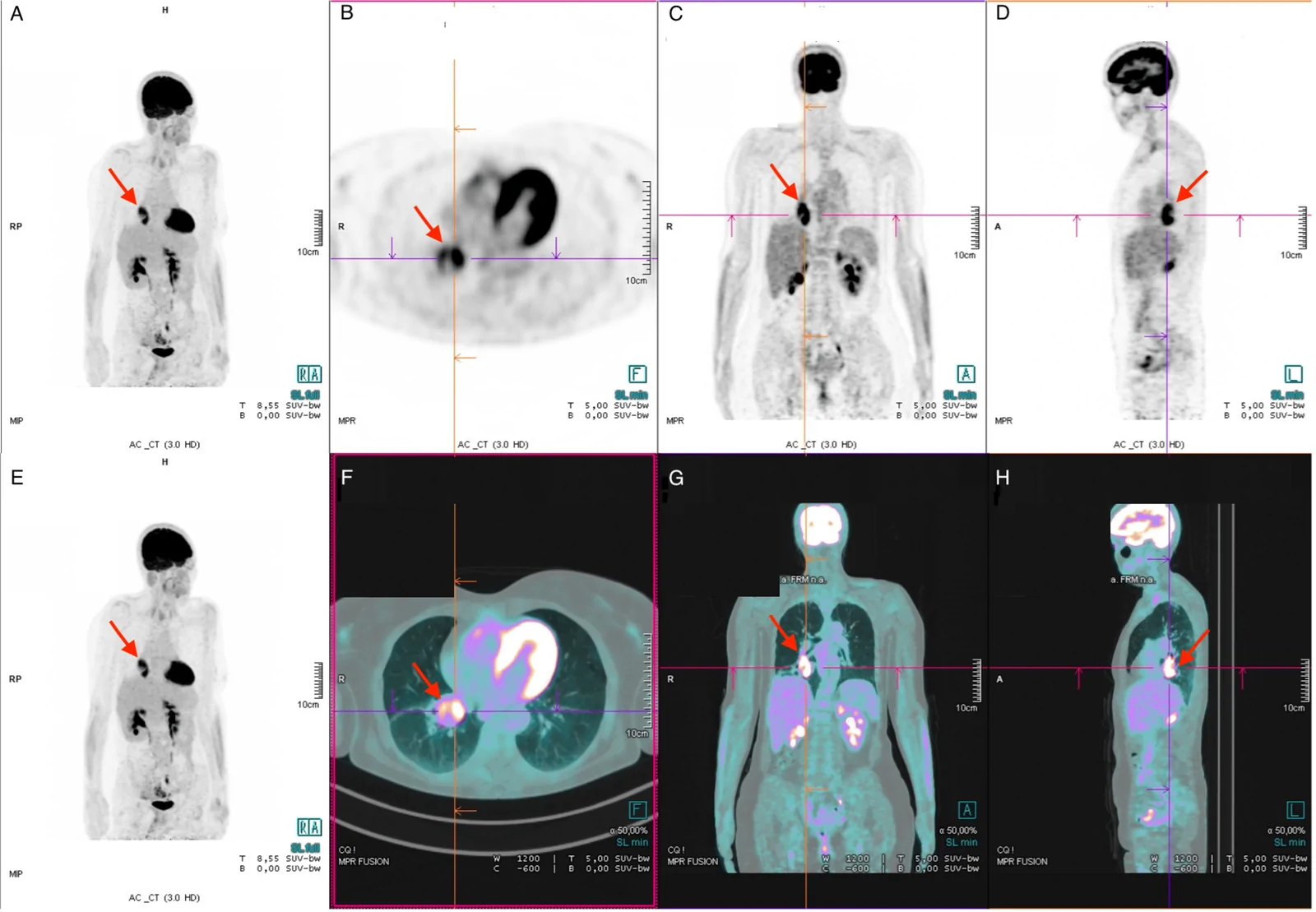
FDG-PET/CT showing metabolic progression of the pulmonary lesion FDG-PET/CT demonstrating metabolic progression of the pulmonary lesion. (A, E) FDG-PET maximum-intensity projection images. (B-D) FDG-PET images in axial, coronal, and sagittal views, respectively. (F-H) Fused FDG-PET/CT images in axial, coronal, and sagittal views, respectively. The arrows indicate the detected lesion described in the text. FDG-PET/CT: fluorodeoxyglucose positron emission tomography/computed tomography

**Figure 5:**
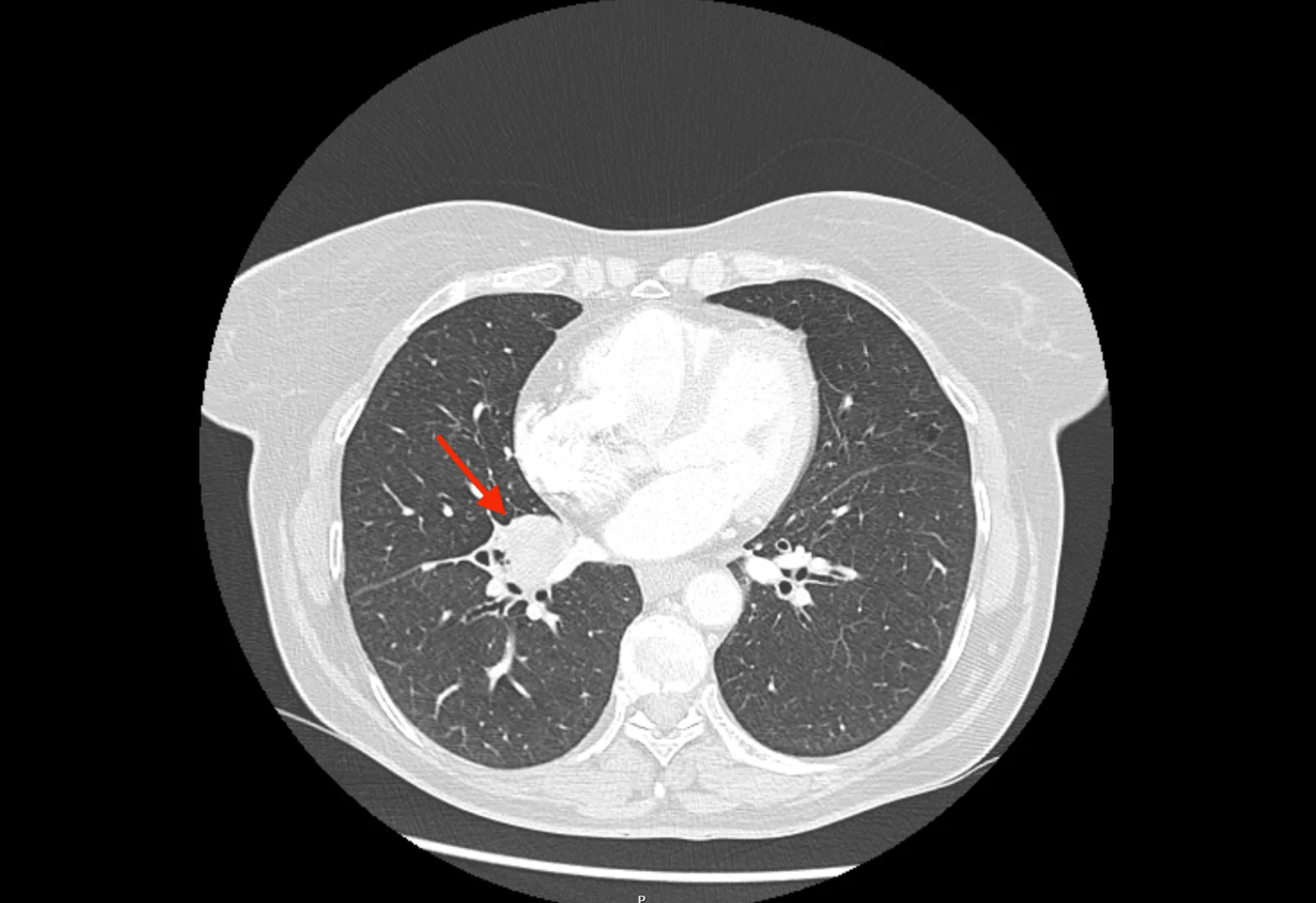
Chest CT confirming progression of the pulmonary lesion Chest CT confirming progression of the pulmonary lesion following immune checkpoint inhibitor therapy. The arrow indicates the detected lesion described in the text. CT: computed tomography

In May 2025, at the first response assessment following SBRT, chest CT demonstrated pulmonary disease progression with new micronodules (Figure 6). Subsequently, in July 2025, systemic palliative chemotherapy with carboplatin area under the curve 5 (AUC 5) and paclitaxel (175 mg/m^2^) was initiated. The patient experienced several toxicities, including grade 2 nausea and vomiting, grade 2 arthralgias and myalgias, and transient grade 2 peripheral neuropathy (sensory with distal distribution), all according to CTCAE v5.0. Due to the severity of the neurotoxicity, the paclitaxel dose was reduced by 25% from the fourth cycle through the sixth and final cycle. At the time of this report, the patient remains in surveillance with stabilized clinical status, ECOG PS performance status 0, and stable disease on imaging.

**Figure 6:**
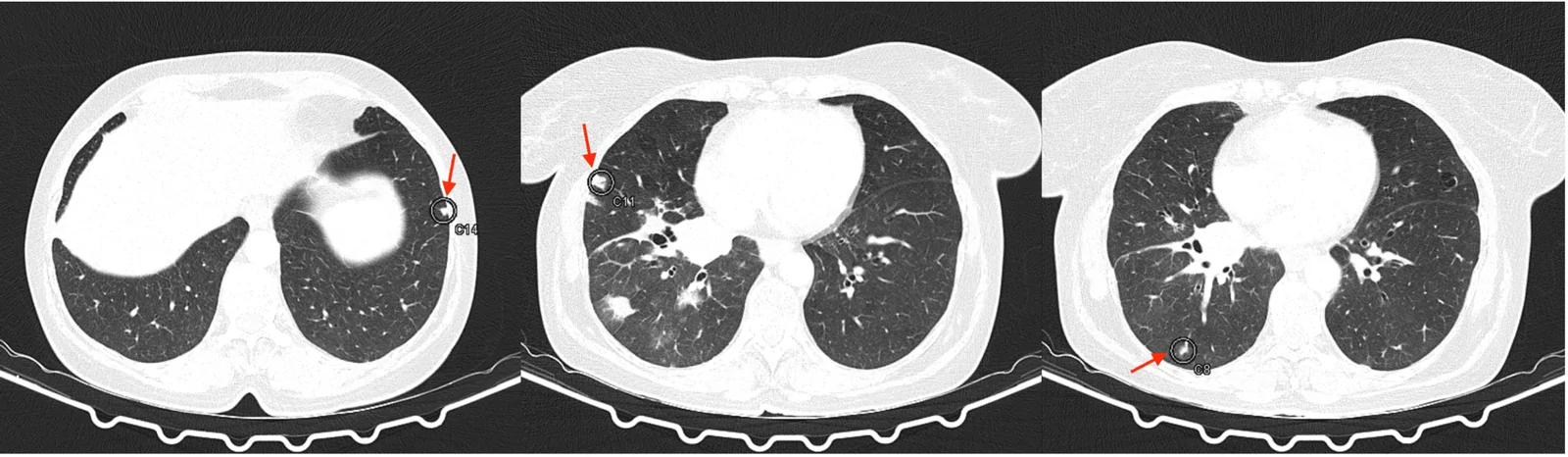
Chest CT showing pulmonary disease progression after SBRT Chest CT performed at the first response assessment following stereotactic body radiotherapy (SBRT), demonstrating pulmonary disease progression with new micronodules. The arrows indicate the lesions described in the text. CT: computed tomography

## Discussion

Metastatic choroidal melanoma exhibits a distinctive dissemination and molecular pattern. While dissemination usually exhibits a marked predilection for the liver, isolated pulmonary metastasis - as observed in this case - represents an uncommon clinical scenario occurring in approximately 10% of patients [1-3]. Importantly, patients with an extrahepatic-only metastatic pattern demonstrate significantly longer survival (median 17 months) compared to those with hepatic (median 11 months) or hepatic-extrahepatic patterns (median 7 months), as demonstrated in a recent retrospective cohort study [4]. Furthermore, mCM is typically characterized by a low TMB and a driver landscape dominated by GNAQ or GNA11 mutations (> 80%) [1,3]. The genomic findings in this case - specifically the TMB of 16.5 mutations/Mb and the somatic NF1 p.R2637* mutation - are atypical for CM and suggest a distinct biological subset [6]. NF1 mutations are rare in CM, occurring in approximately 5% of cases, contrasting with their prevalence in cutaneous melanoma (10-15%) [7]. Interestingly, a study suggested that NF1-wildtype status was associated with longer survival in CM (median 20.8 vs. 7 months), though this finding requires validation in larger cohorts [9].

In this context, the absence of HLA-A02:01 expression precluded the use of tebentafusp - the only Food and Drug Administration (FDA) and European Medicines Agency (EMA)-approved targeted therapy for mCM - which has demonstrated improved overall survival in first-line treatment [2]. Consequently, alternative systemic strategies were required.

High TMB is a validated biomarker for ICI response in several solid tumors. However, its role in mCM is less established due to the overall rarity of high-TMB cases in this histology. Nevertheless, the presence of high TMB provided a strong clinical rationale for the application of dual ICIs in this patient, despite the eventual progression [5,6]. Lack of response despite high TMB highlights the complex interplay between molecular biomarkers and clinical outcomes in mCM, where the immunosuppressive tumor microenvironment may limit ICI efficacy regardless of mutational burden [6].

Additionally, loss-of-function mutations in NF1 (which encodes neurofibromin 1) lead to the hyperactivation of the Rat sarcoma/mitogen-activated protein kinase (RAS/MAPK) signaling pathway. In cutaneous melanoma, NF1-mutant tumors represent a recognized molecular subtype that may respond to mitogen-activated protein kinase (MEK) inhibitors or enrollment in specific MAPK-targeted clinical trials. However, the clinical utility of MEK inhibition in mCM remains investigational, and access to clinical trials was not feasible at the time [6,9].

The management of isolated lung metastasis allowed for a multimodal approach. Local ablative therapy via SBRT was utilized to achieve local control of oligoprogressive disease. While cytotoxic chemotherapy (carboplatin/paclitaxel) is not considered a primary standard for mCM, it remains a palliative option for patients who have exhausted immunotherapy and lack eligibility for tebentafusp [1,7].

## Conclusions

This case highlights the clinical rarity of isolated pulmonary metastasis in CM and the significant role of comprehensive genomic profiling in managing refractory disease. The identification of a high TMB and an NF1 mutation provided biological insights that informed therapeutic sequencing, although the patient did not achieve durable response to immunotherapy. The extrahepatic-only metastatic pattern may have contributed to the relatively favorable clinical course observed.

Additionally, the patient's HLA-A02:01-negative status precluded access to tebentafusp. As precision oncology evolves, the integration of NGS may identify actionable targets and guide the use of ICIs or targeted therapies in atypical mCM presentations. Future research should focus on elucidating mechanisms of immunotherapy resistance in high TMB mCM and exploring the therapeutic potential of MEK inhibition in NF1-mutant disease.
